# Exploring the Impact of In Basket Metrics on the Adoption of a New Electronic Health Record System Among Specialists in a Tertiary Hospital in Alberta: Descriptive Study

**DOI:** 10.2196/53122

**Published:** 2024-04-29

**Authors:** Melita Avdagovska, Craig Kuziemsky, Helia Koosha, Maliheh Hadizadeh, Robert P Pauly, Timothy Graham, Tania Stafinski, David Bigam, Narmin Kassam, Devidas Menon

**Affiliations:** 1 School of Public Health University of Alberta Edmonton, AB Canada; 2 Office of Research Services and School of Business MacEwan University Edmonton, AB Canada; 3 Medicine Department Faculty of Medicine & Dentistry University of Alberta Edmonton, AB Canada; 4 Department of Emergency Medicine Faculty of Medicine & Dentistry University of Alberta Edmonton, AB Canada; 5 Surgery Department Faculty of Medicine & Dentistry University of Alberta Edmonton, AB Canada

**Keywords:** electronic health records, In Basket, metrics, descriptive study, inpatients

## Abstract

**Background:**

Health care organizations implement electronic health record (EHR) systems with the expectation of improved patient care and enhanced provider performance. However, while these technologies hold the potential to create improved care and system efficiencies, they can also lead to unintended negative consequences, such as patient safety issues, communication problems, and provider burnout.

**Objective:**

This study aims to document metrics related to the In Basket communication hub (*time in In Basket per day, time in In Basket per appointment, In Basket messages received per day,* and *turnaround time*) of the EHR system implemented by Alberta Health Services, the province-wide health delivery system called Connect Care (Epic Systems). The objective was to identify how a newly implemented EHR system was used, the timing of its use, and the duration of use specifically related to In Basket activities.

**Methods:**

A descriptive study was conducted. Due to the diversity of specialties, the providers were grouped into *medical* and *surgical* based on previous similar studies. The participants were further subgrouped based on their self-reported clinical full-time equivalent (FTE ) measure. This resulted in 3 subgroups for analysis: *medical FTE <0.5*, *medical FTE >0.5*, and *surgical* (all of whom reported *FTE >0.5*). The analysis was limited to outpatient clinical interactions and explicitly excluded inpatient activities.

**Results:**

A total of 72 participants from 19 different specialties enrolled in this study. The providers had, on average, 8.31 appointments per day during the reporting periods. The providers received, on average, 21.93 messages per day, and they spent 7.61 minutes on average in the *time in In Basket per day* metric and 1.84 minutes on average in the *time in In Basket per appointment* metric. The time for the providers to mark messages as *done* (*turnaround time*) was on average 11.45 days during the reporting period. Although the surgical group had, on average, approximately twice as many appointments per scheduled day, they spent considerably less *connected time* (based on almost all time metrics) than the medical group. However, the surgical group took much longer than the medical group to mark messages as *done* (*turnaround time).*

**Conclusions:**

We observed a range of patterns with no consistent direction. There does not seem to be evidence of a “learning curve,” which would have shown a consistent reduction in time spent on the system over time due to familiarity and experience. While this study does not show how the included metrics could be used as predictors of providers’ satisfaction or feelings of burnout, the use trends could be used to start discussions about future Canadian studies needed in this area.

## Introduction

### Background

Electronic health record (EHR) systems have been implemented with many goals including streamlining information sharing among providers, empowering patients to be active partners in their care, supporting evidence-based individualized care, and monitoring population health. Health care organizations implement EHR systems with the expectation of improved patient care and enhanced provider performance [1,2].

EHR systems are not new in Canada [3]; however, their implementation has been faced with delays, changes in vendors, and reluctant adoption by users [4]. Canada continues to see activity in EHR implementation including in British Columbia [5], Saskatchewan [6], Ontario [7], Alberta (Connect Care; Epic Systems) [8], and Nova Scotia [9].

While EHR systems hold the potential to improve care delivery, they can also contribute to unintended negative consequences, such as patient safety issues, communication problems, and provider burnout [10-13]. Rather than implementing EHR systems and waiting to identify unintended consequences, we should proactively identify metrics to measure the impact of these EHR systems on the work of health care providers and enable ways to improve the diffusion and the subsequent adoption of EHR systems in Canada [9-12].

### Advantages of the EHR System

EHRs are systems designed “to collect patient data in real time to enhance care by providing data at the provider’s fingertips and enabling decision-making where it needs to occur” [14]. These systems provide functions such as viewing (eg, laboratory or test results); documenting (eg, entering data and notes); ordering (eg, referrals, prescriptions, and tests); web-based messaging (eg, notifying patients of test results); care management (eg, disease-specific tools and allergy alerts); analysis and reporting; and patient-directed engagement capabilities (eg, access to own laboratory values and web-based messaging care providers) [15-20]. EHRs can provide benefits such as easy access to accurate and timely point-of-care data, easy navigation to enhance workflow, automation of mundane tasks, evidence-based management pathways to individualize care, convenient sharing of data across organizations, and population health monitoring [13,19,21]. Furthermore, when fully implemented, in some instances, EHRs have resulted in the comprehensive replacement of traditional paper charts [13].

### Burden of EHR System

Although EHR systems are designed to deliver positive outcomes, unintended and technology-specific negative outcomes have also been described related to the workflow, patient-provider interactions (technology seen as impersonal), and the challenges of implementing these technologies within current health care systems [10]. There is increasing awareness that physician well-being has an important impact on the health system, and concerns exist over increasing rates of burnout [11], job dissatisfaction, intention to leave practice, and job turnover [22]. Among the consistently reported drivers of burnout and job dissatisfaction are adverse clinician interactions with EHRs. While EHRs are intended to streamline workflows, they are cited as increasing the inefficiency of clinical work, adding to user frustration [23].

Studies suggest that in clinical environments that include an integrated EHR system, physicians spend an additional 1.5 hours [16] of time using the EHR system for every 1 hour of direct patient interaction, with an additional 6 to 30 hours [17,24] per month of cumulative time spent on EHR documentation and inbox management outside routine working hours [25]. Furthermore, studies have demonstrated a negative impact on providers’ time due to managing test results and communications within In Basket, the EHR system’s communication hub [24,26] for administrative, clerical, and documentation functions and after-hour activities related to accomplishing required tasks [23]. In Basket is where health care providers receive and manage various tasks; web-based messages; and notifications such as appointment requests, medication refill requests, laboratory or imaging results, consultation requests, and web-based messages from other health care team members [27]. Many of these tasks, previously performed by administrative staff, have been increasingly offloaded to providers.

In a primary care setting, a health care provider may receive anywhere from a few to several dozen In Basket messages per day [28]. In specialized settings, such as a hospital or specialized clinic, the volume of In Basket messages can be higher, especially for providers who are involved in complex cases or have a larger patient population [29,30]. Specialists may receive additional types of web-based messages, such as interdepartmental consult requests or referrals from primary care providers. Due to these challenges, the time spent in In Basket activities can vary depending on factors such as the volume of web-based messages, complexity of tasks, and individual work practices [26]. The number of In Basket messages received per day can vary significantly depending on several factors, such as the size of the health care organization, the specialty or department, and the individual provider’s practice [31]. Managing In Basket time requires a balance between efficiency, prioritization, and effective communication to ensure timely and appropriate handling of tasks while delivering quality patient care [26]. Health care organizations have attempted to manage these challenges with one-on-one provider training, optimization and upgrade of processes, increased availability of technical support, added or expanded use of scribes, voice recognition, and improved EHR governance [32,33].

While physician burnout and distress, more broadly, are prevalent issues in Canada [34], to our knowledge, research on EHR use and physician well-being in a Canadian context has been limited, so the relationship of EHRs to surrogate outcomes of well-being (eg, burnout) in the Canadian context is unknown. This knowledge gap is significant, given the substantial disparities between the Canadian and American health care systems, particularly concerning documentation requirements for billing, insurance, and medicolegal purposes, which EHR systems are designed to streamline [32]. Notably, while EHR system implementation is underway in Canadian hospitals, most US hospitals have already adopted these EHR systems [35,36].

Canada operates under a federated system where health governance is federal, but health care delivery and associated tasks such as EHR implementation are managed provincially. Moreover, unlike in the United States, where much of the charting is focused on billing, the Canadian health care landscape differs substantially, as billing is not part of the need for implementing EHR systems [35,36]. These contextual distinctions between the 2 health care systems mean that research findings from one setting cannot simply be extrapolated to the other [13,21,23,24,26,37].

### Objectives

The extent to which EHRs contribute to physician dissatisfaction in Canada, akin to their presumed impact in the United States, remains uncertain. This study lays the groundwork for addressing this gap in knowledge by studying the use metrics of an EHR system implementation in Alberta. This study provides essential insights that not only pave the way for future investigations into the correlation between clinician well-being and EHRs in Canadian contexts but also inform interventional studies aimed at enhancing the user experience. In addition, our findings contribute to the development of best practices for EHR system implementation and use.

This study documented In Basket metrics of an EHR system implemented by Alberta Health Services (AHS), the province-wide health delivery system in Alberta branded as Connect Care. Understanding and documenting granular use metrics of Connect Care in Alberta is foundational for future studies examining the relationship between clinician wellness and EHRs in a Canadian setting, for example, understanding how the EHR-clinician interface contributes to adverse unintended consequences such as burnout. Through gaining an in-depth understanding of how the EHR system in Alberta captures In Basket metrics, this study was designed as a precursor to forthcoming studies examining the association between provider wellness and EHR systems in Canadian settings and to studies focused on improving the EHR system’s user experiences and developing best practices for the EHR system rollout and subsequent use.

## Methods

### Study Design

This was a descriptive study of a volunteer cohort of multidisciplinary specialists working at the University of Alberta Hospital (UAH) [38]. As the goal of the study was to measure the trends in In Basket use in the EHR system from the launch of Connect Care by specialists at the UAH over 33 months of use, this study design allowed us to commence the identification of how the EHR was used, when it was used, and for how long it was used related to the In Basket activities. To the best of our knowledge, this study is the first to explore the In Basket use of Connect Care by specialists in Alberta.

### Explored In Basket Domain Measures (Metrics)

In Basket metrics refer to performance indicators that assess the efficiency and effectiveness of managing tasks, web-based messages, or alerts within the EHR system. These metrics assess various aspects of workflow management and communication within the EHR environment. In this study, we used the following In Basket metrics to capture the use of the In Basket toolbar by study participants: *time spent in the In Basket per day*, *time spent in the In Basket per appointment*, number of *In Basket messages received per day*, and *turnaround Time*. The *time in In Basket per day* metric is defined as the average number of minutes a provider spends in In Basket per day. The *time in In Basket per appointment* metric is the average number of minutes a provider spends in In Basket per scheduled appointment. The *In Basket messages received per day* metric is the average number of In Basket messages a provider receives per day. The *turnaround time* metric is the average number of days a provider takes to mark a message of a specific type as done. Furthermore, In Basket metrics included *appointments per day,* which is the average number of appointments per day within the reporting period for comparison purposes (workload vs use) between the participating specialists.

### Ethics Approval

Ethics approval was received from the University of Alberta Health Research Ethics Board (study ID Pro00119194), and operational approval was received from AHS (OA60778, OA60779, and OA60780).

### EHR System

Connect Care is a comprehensive EHR system that allows users to access, generate, and manage documents, laboratory results, text reports, radiology images, notes, prescriptions, referrals, and web-based messages. Furthermore, Connect Care contains advanced auditing capabilities that record the actions of users when accessing the EHR system.

### Study Setting

AHS is Canada’s largest integrated provincial health system and is responsible for delivering health services to >4.3 million people. Health care programs and services are offered at >900 facilities throughout the province (eg, hospitals, clinics, continuing care facilities, cancer centers, mental health facilities, and community health sites) [39]. The UAH is a quaternary care research and teaching hospital in Edmonton, Alberta. This hospital provides a wide range of inpatient and outpatient diagnostic and treatment services [40]. Study sites within the UAH were selected based on the length of time that they had been using the EHR system. The departments of medicine and surgery at the UAH were part of the first wave of the AHS Connect Care implementation. The specialists in these departments were considered to have used Connect Care for a time period that would provide sufficient use data required for this study.

### Study Sample Recruitment

We decided on the following inclusion criteria for potential study participants: (1) any specialist located at the UAH and (2) ≥7 months of Connect Care use.

We used a purposive sampling method to recruit specialists. The clinical coinvestigators (RPP, DB, and NK) introduced and explained the project at departmental meetings. RPP developed a PowerPoint (Microsoft Corp) presentation, which was adapted by DB and NK to fit the context of their respective departments. During these presentations, the coinvestigators started by describing the potential impact of EHRs on provider well-being, the lack of Canadian use data, the need to understand the user experience, and the opportunity for EHR improvement driven by users. Furthermore, potential participants were informed that their individual results from the study would be shared with them. The clinical leads emailed all attending specialists asking them to complete the consent form (using REDCap [Research Electronic Data Capture]; Vanderbilt University) and provide the required information (eg, department, EHR login ID, clinical workload defined by the self-reported fraction of a full-time equivalent [FTE] measure, and work position) for data access.

### Data Source

The raw In Basket data source was from Signal (an analytical platform developed by Epic Systems Corporation) using EHR user action log data (Epic Systems Corporation, unpublished data, April 2023). The user action log measures the time that the user interacted with the EHR system. The metrics captured in Signal are defined, and quantifiable measurements are used in reports to summarize information about processes or outcomes (Epic Systems Corporation, unpublished data, September 2020). Information about time spent in particular ambulatory (outpatient) In Basket activities (user action logs) was obtained for each participant from their first login to the EHR system. The analysis was limited to outpatient clinical interactions and explicitly excluded inpatient activities.

Once a specialist agreed to participate in the study, their name, login ID, and study ID were stored in a zipped and encrypted file and sent to the AHS Connect Care and Epic data team through REDCap to retrieve the required event logs data. REDCap is a secure web-based platform hosted by the Women and Children’s Health Research Institute in collaboration with the Northern Alberta Clinical Trials and Research Centre at the University of Alberta. Once the Epic data team reviewed the requested information, data were pulled and transferred to the AHS Connect Care team. The anonymized data were zipped and encrypted before being transferred to the principal investigator for analysis.

### Data Description

#### Participants

A total of 72 participants from 19 different specialties enrolled in this study. Of the 72 providers, 1 (1%) provider was excluded due to an absence of In Basket outpatient ambulatory Signal data. Due to the diversity of specialties, the providers were grouped into a *medical group* and a *surgical group* based on previous similar studies and the fact that these categories have similar EHR workflows [41,42].

The participants were further subgrouped based on their self-reported clinical FTE measure. Clinical FTE is a measure used in health care to quantify the work hours of health care providers or clinical staff in relation to a full-time position. This resulted in 3 subgroups for analysis: *medical FTE <0.5*, *medical FTE >0.5*, and *surgical* (all of whom reported FTE >0.5) groups.

In this study, providers in each group are independent of each other (ie, each provider contributes to the weighted means of only 1 group). However, for each In Basket metric, various subsets of providers in the group (ie, medical FTE <0.5, medical FTE >0.5, or surgical group) contribute to the weighted mean of various reporting periods.

#### Missing Values

Once the EHR data for each of the 72 providers was received, we identified missing values. As this study is one of the first to explore the provider’s use of Connect Care in the Alberta context, we wanted to gain an in-depth understanding of the missing In Basket outpatient ambulatory provider-related Signal data.

On the basis of discussions with the Epic team, the study team identified 3 reasons for missing values in the data. The first reason was that a participant must be “registered” with Connect Care (AHS), be active, and must have logged in to the EHR system and seen at least 1 patient in the reporting period [38]. Second, for the *time in In Basket per appointment* metric, there was an additional inclusion criterion where the provider needed at least 5 appointments scheduled per week within the reporting period for Signal to capture user interactions in the EHR system [2]. We identified this as an issue as many part-time specialists might have ≤4 appointments per week; for example, if they were on ward duties, they would be managing only inpatients during that time. Although they interacted with the EHR system, no data would be recorded for these metrics. Since inpatient data were not studied, the true impact of EHR system use might be underestimated. The third reason for missing data is that the EHR system did not capture any data for certain metrics for all participating providers during certain months such as the *In Basket messages* *received per day* (missing data for all providers during April 2021, May 2021, July 2021, August 2021, and September 2021) and *time in In Basket per appointment* (April 2021) metrics. Neither we nor the analysts from Epic Systems could determine the root cause of the missing data.

On the basis of these findings, we used a complete case analysis to address missing values [29]. The observations with denominator=0 were excluded. The weighted averages did not capture the missing values data. As each In Basket metric was considered individually, a provider had to have at least 1 month of data for a particular In Basket metric to be included in the metric analysis.

### Data Ranges

The start date was November 1, 2019 (the date of launch of Connect Care), and the end date was July 30, 2022, for the In Basket metrics.

### Data Types

Depending on the available data for the metrics, the monthly reporting periods included in the analysis ranged between 14 and 33 months. The overall amount of data varied between 1528 (15.92%) observations for the *time in In Basket per appointment* metric and 2203 (22.95%) observations for the *time in In Basket per day* metric. The total number of observations for all included metrics was 9598.

### Statistical Analysis

Data aggregation, analysis, and visualization were performed using SAS (version 9.4; SAS Institute) and Tableau (version 2021.4.3; Tableau Software, LLC) [43,44]. The numerator and denominator from each metric were used to calculate the weighted daily means of all participants and each group.

A 2-sample *t* test (2-tailed) was used to compare the weighted daily mean of every metric for the medical FTE >0.5 group with the medical FTE ≤0.5 group and compare those of the medical FTE >0.5 group with the surgical FTE >0.5 group. A weighted average calculates the mean of a data set while considering the varying importance or significance of each number within the set. This approach is commonly used in statistical analysis. It is a critical tool for addressing fluctuations, managing uneven or distorted data, and ensuring fair representation of similar data points based on their respective weights.

In time-series analysis, such as the one we have conducted, time-weighted averages were used because the time series was not evenly sampled. Ideally, data points in a time series are evenly spaced, such as hourly, daily, or monthly intervals, where each point carries equal weight. However, in our data set, reporting periods were irregular, with varying lengths ranging from 27 to 35 days. Consequently, these reporting periods had different weights. To address this, we converted the reporting periods to a daily scale, ensuring each data point carried equal weight. In summary, a time-weighted average assigns weight to each value based on its duration relative to surrounding points, leading to significantly improved accuracy in the final calculation.

Trend analysis was used to evaluate the use trends over time to determine changes in Connect Care use by the participating providers. A simple moving average (SMA) curve was used to explore the learning curves (changes over time) for each metric [45,46]. A linear trend line was fitted to the SMA curve for each group (ie, medical FTE <0.5, medical FTE >0.5, and surgical groups) based on each included metric to determine the changes in trends (ie, whether the slope increased, decreased, or remained unchanged).

In all these analyses, a *P* value of <.05 was considered statistically significant.

## Results

### Participant Characteristics

In total, 71 providers were included in the analysis. Of the 71 providers, 29 (40%) were women providers and 43 (60%) were men providers. The analysis did not compare results by age or gender because the numbers were small. The largest specialty group was internal medicine (n=14, 20%), followed by nephrology (n=10, 14%) and general surgery (n=9, 13%). The least represented specialties were dermatology, intensive care, neurosurgery, and cardiac surgery, at about 1% (n=1) each. Due to the diversity of specialties, the providers were grouped into a medical group (n=53, 75%) and a surgical group (n=18, 25%) based on previous similar studies (Multimedia Appendix 1) [41,42].

Furthermore, the self-reported FTE was used to further subgroup participants. Of the 53 participants in the medical group, 27 (51%) participants reported FTE <0.5 and 26 (49%) participants reported FTE >0.5. All 18 (100%) surgical specialists reported FTE >0.5. This resulted in 3 subgroups: medical FTE <0.5, medical FTE >0.5, and surgical (all FTE >0.5) groups.

### Overall Results

Table 1 shows the weighted daily means for all participating providers (including weighted daily means for the medical and surgery groups) for each metric in this study. The use of weighted daily means indicates a more precise method for determining the average appointments per day compared to a simple average based solely on the number of providers and reporting periods. In this study, because the reporting periods varied in duration, they were assigned different weights based on the number of days within each period. This adjustment ensured a more accurate representation of daily appointment averages.

**Table 1:** Weighted daily means per metric for the medical and surgical groups.

Metric	Weighted daily mean (95% CI)		
	All specialties	Medical group	Surgical group
Appointments per day	8.31 (8.27-8.35)	6.41 (6.39-6.44)	14.01 (13.93-14.10)
In Basket messages received per day	21.93 (21.64-22.22)	22.02 (21.67-22.38)	21.70 (21.45-21.94)
Time spent in In Basket per day	7.61 (7.59-7.64)	8.86 (8.84-8.89)	3.95 (3.92-3.97)
Time spent in In Basket per appointment	1.84 (1.83-1.85)	2.78 (2.77-2.79)	0.60 (0.59-0.61)
Turnaround time	11.45 (10.83-12.08)	9.72 (9.21-10.23)	16.22 (14.69-17.76)

On the basis of the weighted daily means adjustment, each provider had, on average, 8.31 appointments per day during the entire reporting period. The providers received, on average, 21.93 web-based messages per day and spent 7.61 minutes on average in the *time in In Basket per day* metric and 1.84 minutes on average in the *time in In Basket per appointment* metric. The time for the providers to mark messages as “done” (meaning that they had completed tasks associated with them; Turnaround Time) was, on average, 11.45 days during the reporting period. Although the surgical group had, on average, approximately twice as many appointments per scheduled day, they spent considerably less “connected time” (based on almost all time metrics) than the medical group. However, the surgical group took much longer than the medical group to mark messages as done (Turnaround Time; Table 1).

Table 2 shows the weighted daily means per provider group (ie, medical FTE <0.5, medical FTE >0.5, and surgical groups) for each metric in this study. According to the raw data, the medical FTE <0.5 and the surgical groups had, on average, more appointments per day during the reporting period than the medical FTE >0.5 group. In addition, all the time metrics indicate that the medical FTE <0.5 group had less time on Connect Care than the medical group FTE >0.5. The same was observed between the medical FTE >0.5 and the surgical groups, except for the *turnaround time* metric (Table 2).

**Table 2:** Full-time equivalent (FTE) comparison between medical and surgical groups.

Metric	Weighted daily mean (95% CI)		
	Medical group FTE ≤0.5	Medical group FTE >0.5	Surgical group
Appointments per day	6.47 (6.44-6.49)	6.36 (6.33-6.39)	14.01 (13.93-14.10)
In Basket messages received per day	20.59 (20.39-20.78)	22.07 (21.75-22.38)	21.70^a^ (21.45-21.94)
Time spent in In Basket per day	7.98 (7.95-8.02)	9.73 (9.68-9.77)	3.95 (3.92-3.97)
Time spent in In Basket per appointment	2.69 (2.68-2.71)	2.88 (2.86-2.90)	0.60 (0.59-0.61)
Turnaround time	6.23 (5.98-6.48)	12.24 (11.50-12.98)	16.22 (14.69-17.76)

^a^Surgical group versus medical FTE >0.5 group comparison: *P* value=.07.

### Trend Analysis

Table 3 presents the results of the trend analysis. All 3 groups had a statistically significant increase in the appointments per day and turnaround time metrics over the study period.

As presented in Table 3, for the medical FTE ≤0.5 group, the *appointments per day*, *In Basket messages received per day*, *time in In Basket per appointment*, and *turnaround time* metrics showed statistically significant changing slopes (increasing trends over time), while the *time in In Basket per day* metric remained unchanged. The largest slope for this group was observed for the *turnaround time* metric with a value of 0.0055.

For the medical FTE >0.5 group, all metrics showed statistically significant changes (Table 3). This group showed the largest number of statistically significant trend changes among the 3 studied groups. A total of 3 metrics (ie, *appointments per day*, *In Basket messages received per day*, and *time in In Basket per day*) that showed statistically significant changes had increasing trends, while the *time in In Basket per appointment* metric showed statistically significant changes with a negative slope (decreasing trend).

**Table 3:** Trend analysis over time for the 3 groups: medical full-time equivalent (FTE ) <0.5, medical FTE >0.5, and surgical groups.

Metric	Medical FTE <0.5 group			Medical FTE >0.5 group				Surgical group		
	Slope	*P* value	Trend	Slope	*P* value	Trend	Slope		*P* value	Trend
Appointments per day	0.0008	<.001	*Increasing^a^*	0.0014	<.001	*Increasing*	0.0015		<.001	*Increasing*
In Basket messages received per day	0.0047	<.001	*Increasing*	0.0194	<.001	*Increasing*	−0.0020		.17	Unchanged^b^
Time spent in In Basket per day	0.0002	.16	Unchanged	0.0009	<.001	*Increasing*	−0.0004		.01	*Decreasing^c^*
Time spent in In Basket per appointment	0.0003	<.001	*Increasing*	−0.0006	<.001	*Decreasing*	−0.0001		<.001	*Decreasing*
Turnaround time	0.0055	<.001	*Increasing*	0.0147	<.001	*Increasing*	0.0175		.001	*Increasing*

^a^Increasing: positive slope and *P* value is statistically significant.

^b^Unchanged: *P* value is not statistically significant.

^c^Decreasing: negative slope and *P* value is statistically significant.

For the surgical group, the *appointments per day* and *turnaround* *time* metrics showed a statistically significant increasing trend, while the *time in In Basket per day* and *time in In Basket per appointment* metrics showed a statistically significant decreasing trend.

Although there were increasing and decreasing patterns among the included metrics, there were no obvious patterns across metrics and among groups. Therefore, there does not seem to be evidence of a “learning curve,” which would have shown a consistent reduction in time spent in the EHR system over time due to familiarity and experience.

### Findings by Metric

The following sections describe the findings for each metric.

#### Appointments Per Day

During the reporting period, the weighted daily average number of appointments per day was 8.31 (95% CI 8.27-8.35) for all providers. For the medical group, the daily weighted average was 6.41 (95% CI 6.39-6.44), while for the surgical group, this number was 14.01 (95% CI 13.93-14.10) appointments per day. The weighted daily mean for the medical FTE ≤0.5 group (mean 6.47, 95% CI 6.44-6.49), compared to the mean for the medical FTE >0.5 group (mean 6.36, 95% CI 6.33-6.39), was significantly different (Multimedia Appendix 2).

Although the slope changes were subtle, the SMA trends for the *appointments per day* metric for all 3 groups were statistically increasing over time (Multimedia Appendix 2).

#### In Basket Messages Received Per Day

The weighted daily mean of web-based messages received was 21.93 (95% CI 21.64-22.22) messages for all 71 providers. The weighted daily mean for the medical FTE >0.5 group was significantly larger than that for the medical FTE ≤0.5 group. Furthermore, the difference between the weighted daily mean values of the medical FTE >0.5 group (mean 23.29, 95% CI 22.70-23.57) and the surgical group (mean 21.70, 95% CI 21.45, 21.94; *P*<.001) was statistically significant (Multimedia Appendix 2).

In June 2021, Signal data recorded that 1 particular specialist received an unusually large number of In Basket messages. After an examination, it was determined that this was due to the EHR system sending a batch of all laboratory results from many patients to this particular medical specialist, who was probably on call. The spike from this individual’s data is reflected in the 2 graphs related to the medical group (FTE >0.5) and the graph for all providers (Multimedia Appendix 2). While this particular case may be seen as an outlier, it serves as an illustration of what can potentially happen within an EHR system. Instances like this one may not be uncommon.

According to the SMA trend analysis, both medical groups experienced statistically significant increasing trends in this metric, while the surgery group’s trend remained statistically unchanged. The trend change was much more pronounced for the medical FTE >0.5 group (slope=0.0194) than that of the medical FTE ≤0.5 group (slope=0.0047). Notably, for the medical FTE >0.5 group, this metric had the largest slope and was the fastest changing over time (Multimedia Appendix 2). This might have been because of the “anomaly” of a single physician in the medical FTE >0.5 group receiving a very large number of emails, as described in the previous paragraph. Furthermore, these results show the situations that are possible within the EHR system and need to be recognized.

#### Time in In Basket Per Day

The weighted daily mean for all providers was 7.61 (95% CI 7.59-7.64) minutes in In Basket per day. The weighted daily mean for the medical group was 8.86 (95% CI 8.84-8.89) minutes in In Basket per day, while that for the surgical group was 3.95 (95% CI 3.92-3.97) minutes per day. The medical FTE ≤0.5 group’s weighted daily mean was 7.98 (95% CI 7.95-8.02) minutes in In Basket per day, and the weighted daily mean for the medical FTE >0.5 group (*P*<.001) was 9.73 (95% CI 9.68-9.77) minutes per day. The surgical group spent less time in In Basket per day than the medical FTE >0.5 group (mean 3.95, 95% CI 3.92-3.97, vs mean 9.73, 95% CI 9.68-9.77; *P*<.001; Multimedia Appendix 2).

On the basis of the trend analysis, the medical FTE >0.5 group showed a statistically significant increasing trend for this metric, while the surgery group showed a statistically significant decreasing trend and the medical FTE ≤0.5 group’s trend stayed statistically unchanged. While the result of trend analysis for this metric is different for each group, it is important to note that the slopes for each group were very small and clinically insignificant (Multimedia Appendix 2).

#### Time in In Basket Per Appointment

After analyzing 1528 observations related to the time that providers spent in the In Basket per appointment, the total average time for both surgical and medical groups was 1.84 (95% CI 1.83-1.85) minutes. The surgical groups spent 0.60 (95% CI 0.59-0.61) minutes, while the medical group spent 2.78 (95% CI 2.77-2.79) minutes. The weighted daily mean for the medical FTE ≤0.5 group was significantly different compared to the mean for the medical FTE >0.5 group (mean 2.69, 95% CI 2.68-2.71, vs mean 2.88, 95% CI 2.86-2.90; *P*<.001). Furthermore, a significant difference was observed when comparing the medical FTE >0.5 group (mean 2.88, 95% CI 2.86-2.90) and the surgical group (mean 0.60, 95% CI 0.59, 0.61; *P*<.001; Multimedia Appendix 2).

For the *time in In Basket per appointment* metric, the medical FTE ≤0.5 group was the only group that saw a statistical increase in their use over time. The other 2 groups showed a statistical decrease in their use over time for this metric (Multimedia Appendix 2).

#### Turnaround Time

*Turnaround time* is a metric group under the In Basket category within Signal. It reports the average number of days a provider takes to mark a message of a specific type as “done.” According to the data, the surgical group spent 16.22 (95% CI 14.69-17.76) days on average to mark messages as done. The medical group spent, on average, 9.72 (95% CI 9.21-10.23) days to mark messages as done (Multimedia Appendix 2). For this metric, a significant difference was observed when comparing the 2 medical groups and between the medical FTE >0.5 and the surgical group. The study team was unable to identify the reasons for the delays.

For this metric, spikes in recorded data were observed (Multimedia Appendix 2) for 2 study participants (1 medical and 1 surgical specialist) over several months, indicating extremely long delays in marking received messages as “done.” An explanation for these anomalies in data capture within the *turnaround time* metric remains elusive. Once more, we encounter an outlier; nonetheless, it serves as an example of potential EHR system use scenarios.

On the basis of the SMA trend analysis, all 3 groups experienced statistically increasing trends over time for their *turnaround time* metric (Multimedia Appendix 2). The largest slope (0.0175) belonged to the surgical group and the smallest slope (0.0055) belonged to the medical FTE ≤0.5 group for this metric.

## Discussion

### Principal Findings

Implementing Connect Care by AHS has transformed how providers capture and share information by establishing changes to workflows, processes, and charting approaches [47]. While the overall objective is to establish uniformity in the EHR system’s use, this study has revealed disparities in the timing of task completion within the EHR system. Furthermore, in certain cases, outliers have emerged whose use patterns are not easily explained with the existing data. This study revealed significant gaps in our understanding of EHRs and In Basket management, highlighting the need for further exploration and comprehension in these areas.

Khairat et al [48] evaluated the time spent by general and specialist pediatricians performing clinical documentation and In Basket tasks outside work hours. Specialists spent more time in the EHR system, and “this may be because specialists see more complex patients and, therefore, need more time to review the patient chart and to respond to In Basket messages” [48]. Although in our study, we cannot say what percentage of workload the providers spent on In Basket activities, we identified that they spent 7.61 minutes in the *time in In Basket per day* metric and 1.84 minutes in the *time in In Basket per appointment* metric*.* According to the raw data, the medical FTE <0.5 group and the surgical group had, on average, more appointments per day during the reporting period than the medical FTE >0.5 group. It would be valuable to explore the main workflow drivers of In Basket time and try to optimize efficiency in this area for all specialties.

The proportion of time spent in the EHR system based on the included metrics between the providers within the medical groups (FTE ≤0.5 and FTE >0.5) was similar; however, little can be concluded about the similarities or differences in use due to the high variability within the specialties. Although data analysis showed statistical significance for all metrics, it is apparent that FTE made no difference to the workload between providers working (FTE ≤0.5 or FTE >0.5). Before comparing part-time medical providers with full-time ones, we could not definitively attribute the observed differences between the medical and surgical groups to the fact that some medical providers worked part time or to the fact that all surgical providers worked full time. Our study did not reveal important differences in In Basket metrics among medical specialists regardless of the clinical FTE . Significant differences were observed between medical and surgical colleagues. Presumably, these differences relate to broad differences in medical versus surgical consultation and their associated workflows.

When comparing the 3 groups, the medical FTE >0.5 group was more “connected” than the medical FTE <0.5 group and the surgical group when considering the *time in In Basket per day* and *time in In Basket per appointment* metrics. Although the surgical group treated more patients (on average, 14.01 appointments during the reporting period), they spent less time in In Basket per day and per appointment, so they were “less connected” than the 2 medical groups. Nonetheless, while the data do not provide a direct explanation for these differences, they do provide insight into the structuring of workloads. This insight is crucial for comprehending how various professionals use the EHR system and identifying areas where workflow enhancements could prove beneficial.

We identified several providers’ data that were outliers in terms of their EHR use. For example, 2 providers took inordinately long times to mark received web-based messages as *done* (*turnaround time*), which impacted the data on between-group differences. Furthermore, there was a medical provider who received an extremely high number of web-based messages in June 2021. Such outliers demonstrate that certain scenarios can significantly influence the averages of various metrics, leading to skewed results. It underscores the possibility that data generated by the EHR system may not always be accurate, emphasizing the need for discussions and considerations with EHR system vendors regarding EHR functionality and measures to reduce outlier occurrences. Future research with a more robust statistical approach should be conducted to delve deeper into addressing and mitigating anomalies in the data.

One factor that we identified in our study is that Connect Care did not capture all interactions due to various vendor-imposed rules (eg, 5 appointments per reporting period). Similarly, Cohen et al [49] identified issues with vendor-derived metrics and how different vendors calculated the same activities in different ways and identified that not all EHRs (vendors) drew information from audit log data, which led to the inability to provide the whole picture of provider’s interaction with the EHR system [49]. Therefore, using only vendor-derived metrics may miss important aspects of the true impact of the EHR system on users. In the study by Cohen et al [49], 1 participant stated that “if different EHR (vendors) are attacking the issue differently, you will get variations not related to burden but just how the math is done.” Documentation time for In Basket use must be captured completely with the intent to understand how In Basket contributes to the overall workload of providers. If EHR systems are being associated with burnout, In Basket messages could be a starting point for common ground around the discussion of how web-based messages should be delivered and managed [50].

### Future Directions

On the basis of the results from this study, we identified several future studies that can build upon this study. This study was descriptive and did not explore the correlation between the included metrics and provider satisfaction and burnout due to EHR system use. The next step would be to conduct a study exploring the circumstances around the individual EHR data. It would be valuable to explore the main workflow drivers of In Basket use time and try to optimize efficiency in this area for all specialties. A qualitative study should be conducted to explore the variances between actual and perceived EHR system use. While data from this study do not provide a direct explanation for these differences, they do provide insight into the structuring of workloads.

Furthermore, future studies should focus on the difference between providers with part-time and full-time clinical schedules and how that translates into EHR use. This insight is key for understanding how various professions use the EHR system in order to identify areas where workflow enhancements could prove beneficial. Moreover, future research should explore EHR use between different specialties and whether these specialties impact EHR use habits. In addition, studies should explore the association between other metrics and quality outcomes. Finally, future studies need to work on developing strategies for EHR data quality appraisal. In our study, we identified that the data generated by the EHR system may not always be accurate, emphasizing the need for discussions and considerations with EHR vendors regarding EHR functionality. In future studies with a more robust statistical approach, there may be an opportunity to delve deeper into addressing and mitigating anomalies in the data.

### Limitations

This study has several limitations. The analyzed data were only the participating providers’ ambulatory (outpatient) data. Inpatient data were not included, which might have provided additional information on some of the metrics (inpatient data were unavailable for all included metrics in this study). Another limitation is the underestimation of some metrics based on how Epic defines and captures activity (eg, a provider needs at least 5 appointments scheduled per week within the reporting period and inbox activities related to phone calls or chart review). Furthermore, to address some In Basket issues, a person may need to access other parts of the EHR system to gather more information or complete some other task (eg, write a prescription) and only then go back into the In Basket to sign off on it. Therefore, the actual time in the In Basket is a systematic underestimation of the actual time it took to complete a task.

Due to the high variability of specialties (19 in total) and the low number of recruited providers for each specialty (ranging between 1 and 14 providers), we were unable to explore and compare the differences in EHR use between the specialties. The small number of participants might have created a bias regarding the reasons for participation. Another study limitation was that the FTE was self-reported, which might have led to providers over- or underestimating their clinical schedules. The final limitation is that we did not evaluate the types of web-based messages that the providers received in the In Basket. As this is one of the first studies evaluating Connect Care, we deemed that the focus should be on the overall metrics rather than the submetrics or categories.

### Conclusions

This study demonstrated the enormous promise of the ability to harvest data from an EHR that describes system use and the potential impact that it has on the workflow of physicians. To take complete advantage of this, there must be an appropriate understanding of how EHR systems capture and measure the use by providers. This would be foundational to forthcoming studies examining the association between provider wellness and EHR systems in Canadian settings and studies focused on improving the EHR system’s user experiences, developing best practices for EHR systems rollout and subsequent use, and understanding how the interface of the user and the EHR systems interrelate. Although this study does not show how the included metrics could be used as predictors of providers’ satisfaction or feeling of burnout, the use trends could be used to start discussions about future Canadian studies needed in this area.

## Appendix

Multimedia Appendix 1 Grouping of included providers according to specialties.

Multimedia Appendix 2 Findings by metric.

## Abbreviations

AHS: Alberta Health Services
EHR: electronic health record
FTE: full-time equivalent
REDCap: Research Electronic Data Capture
SMA: simple moving average
UAH: University of Alberta Hospital
