# Supplementary material for: Exploring the Impact of In Basket Metrics on the Adoption of a New Electronic Health Record System Among Specialists in a Tertiary Hospital in Alberta: Descriptive Study
Source: J Med Internet Res. 2024 Apr 29;26:e53122. doi: 10.2196/53122 (PMC11091810; doi:10.2196/53122)
Supplement: Multimedia Appendix 1 [file jmir_v26i1e53122_app1.docx]

**Multimedia Appendix 1**

# Grouping of included providers according to specialties

| Table 2. Grouping of included providers according to specialties. | | | |
| --- | --- | --- | --- |
| **Medical Group** | **Number of providers** | **Surgery Group** | **Number of providers** |
| Internal medicine | 14 | General Surgery | 9 |
| Nephrology | 10 | Orthopedic Surgery | 3 |
| Neurology | 7 | Plastic surgery | 2 |
| Infectious Diseases | 3 | Urology | 2 |
| Hematology | 3 | Cardiac Surgery | 1 |
| Physical Medicine and Rehabilitation | 3 | Neurosurgery | 1 |
| Respirology | 3 |  |  |
| Cardiology | 2 |  |  |
| Geriatrics | 2 |  |  |
| Endocrinology | 2 |  |  |
| Gastroenterology | 2 |  |  |
| Dermatology | 1 |  |  |
| Intensive care | 1 |  |  |
| **Total** | **53** |  | **18** |
