# Supplementary material for: Exploring the Impact of In Basket Metrics on the Adoption of a New Electronic Health Record System Among Specialists in a Tertiary Hospital in Alberta: Descriptive Study
Source: J Med Internet Res. 2024 Apr 29;26:e53122. doi: 10.2196/53122 (PMC11091810; doi:10.2196/53122)
Supplement: Multimedia Appendix 2 [file jmir_v26i1e53122_app2.docx]

**Multimedia Appendix 2**

# Findings by metric

## Appointments per Day


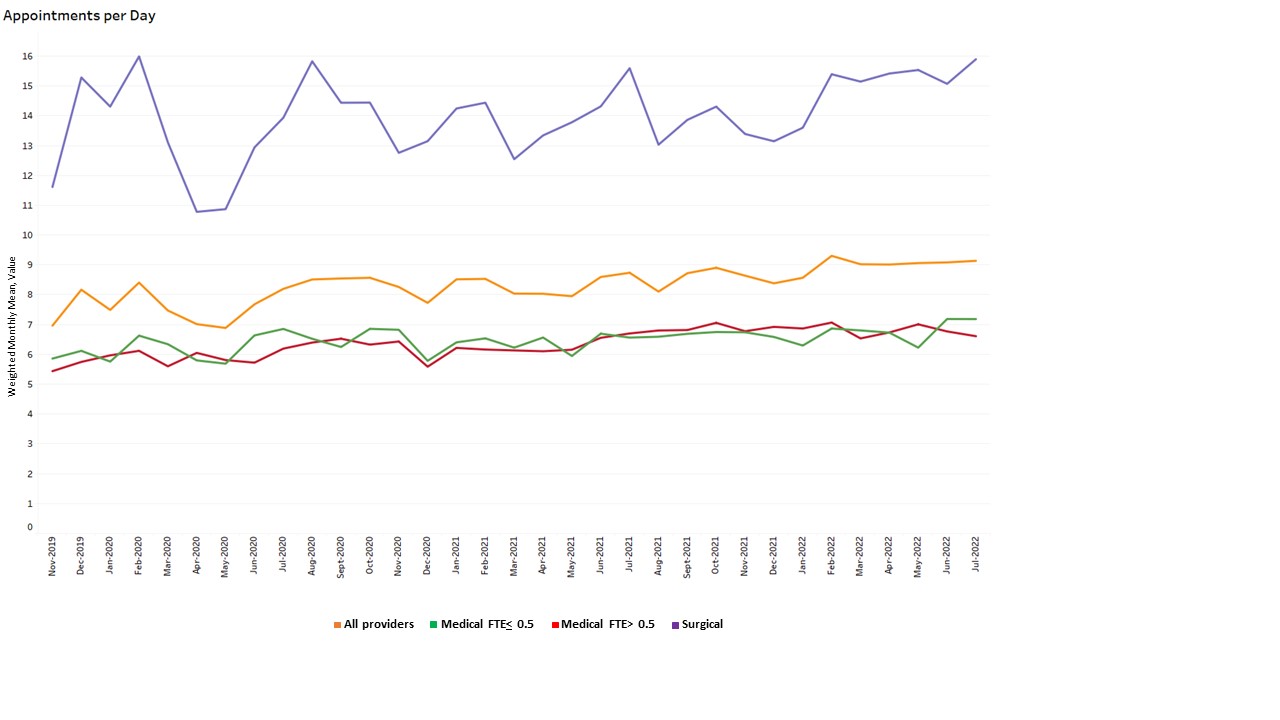


**Figure 1**. Appointments per Day (group mean values).


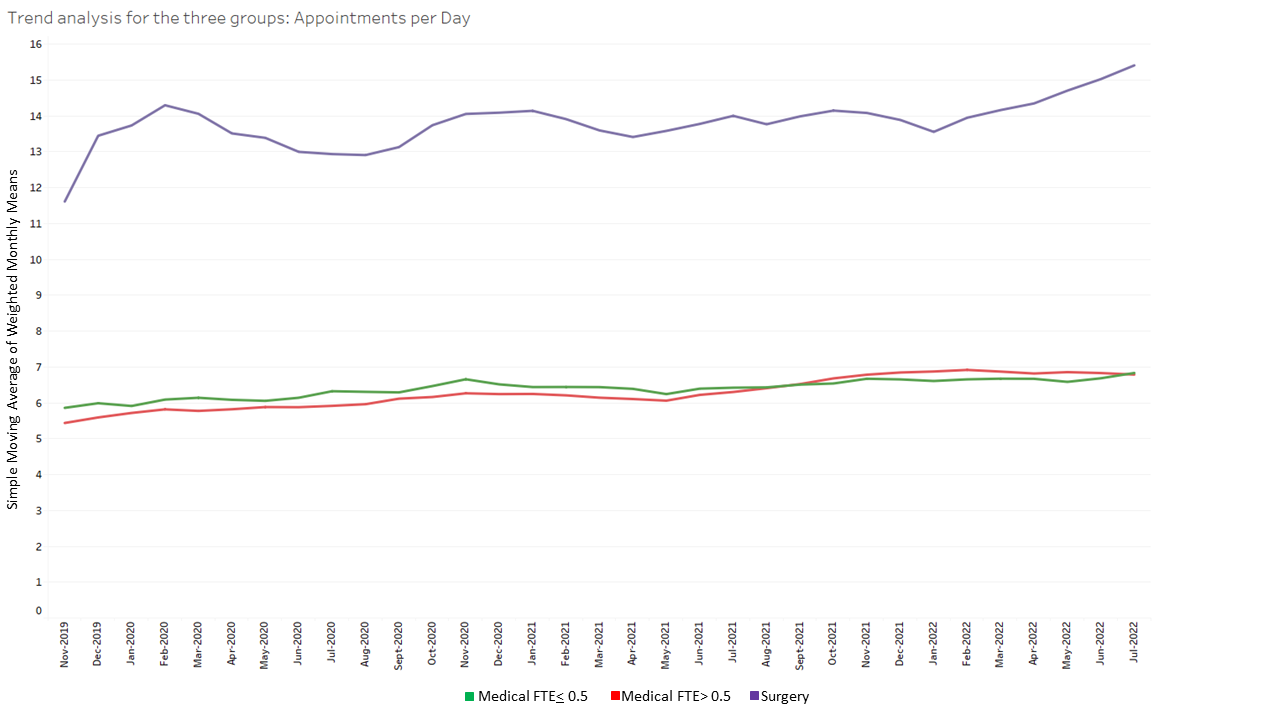


**Figure 2**. Appointments per Day (SMA analysis by group).

## In Basket Messages Received per Day


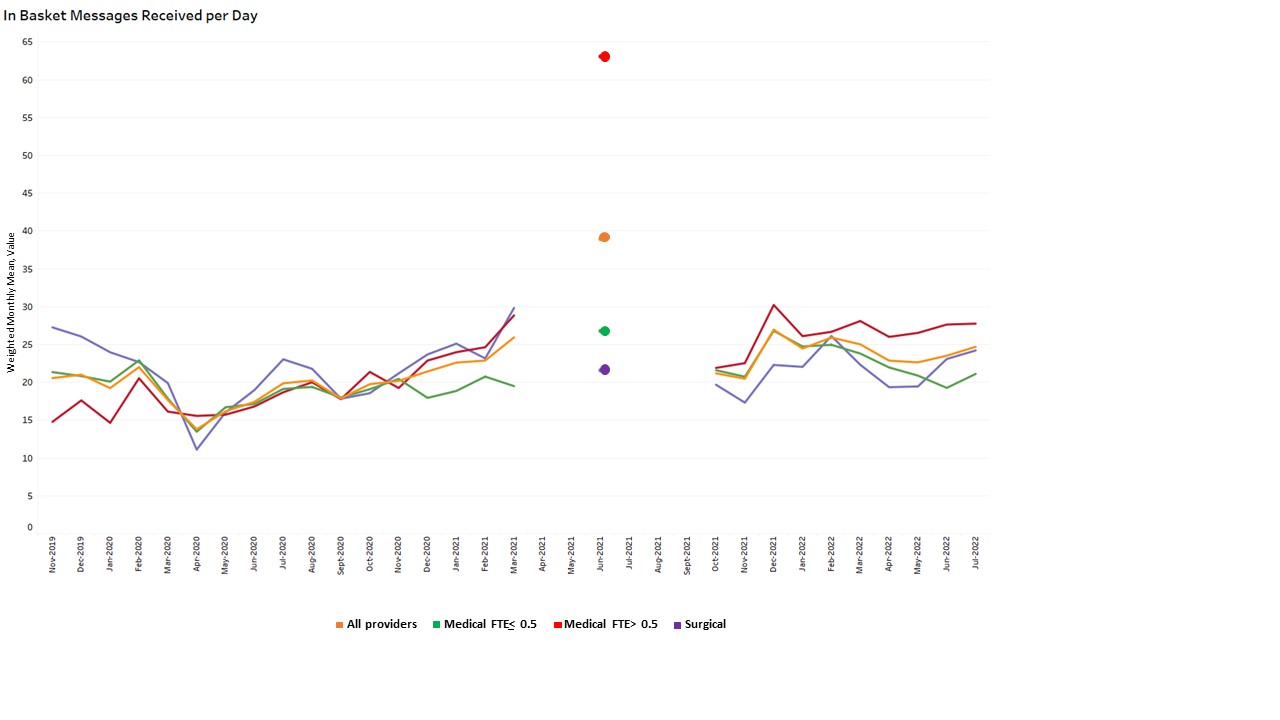


**Figure 3**. In Basket Messages Received per Day (group mean values).


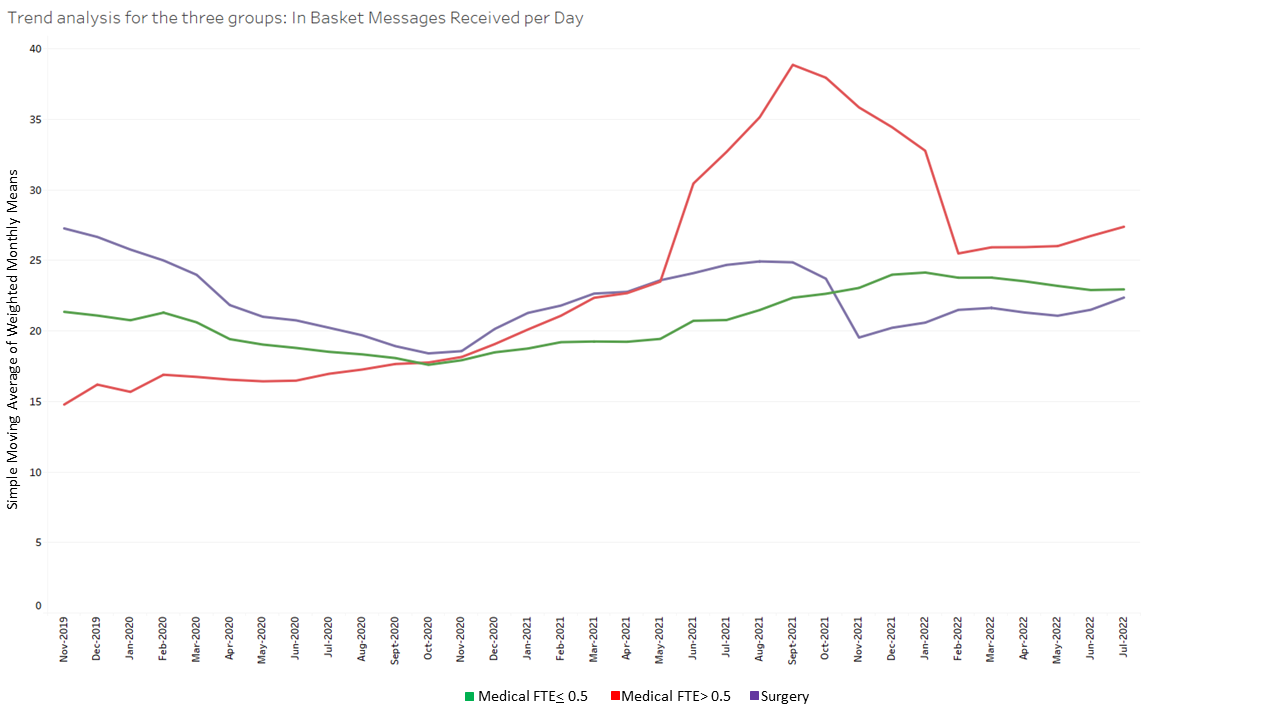


**Figure 4***.* In Basket Messages Received per Day (SMA analysis by groups).

## Time in In Basket per Day


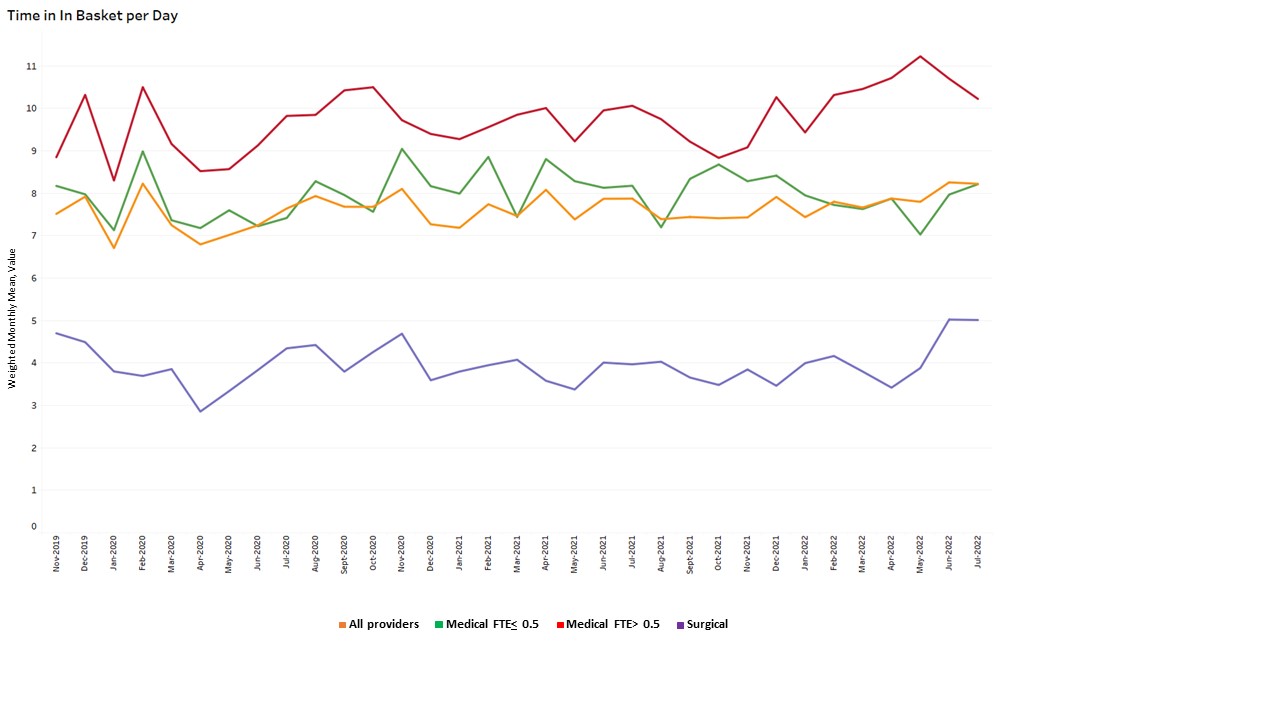


**Figure 5***.* Time in In Basket per Day (group mean values).


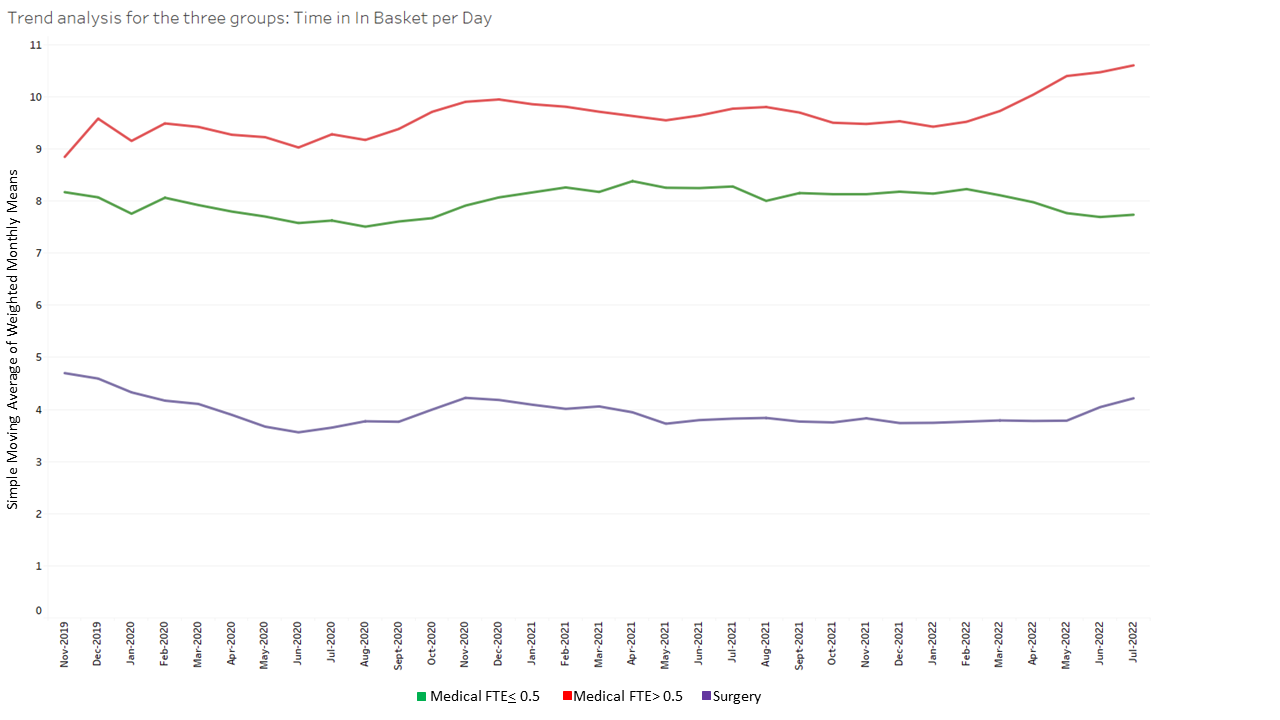


**Figure 6**. Time in In Basket per Day (SMA analysis by group).

## Time in In Basket per Appointment


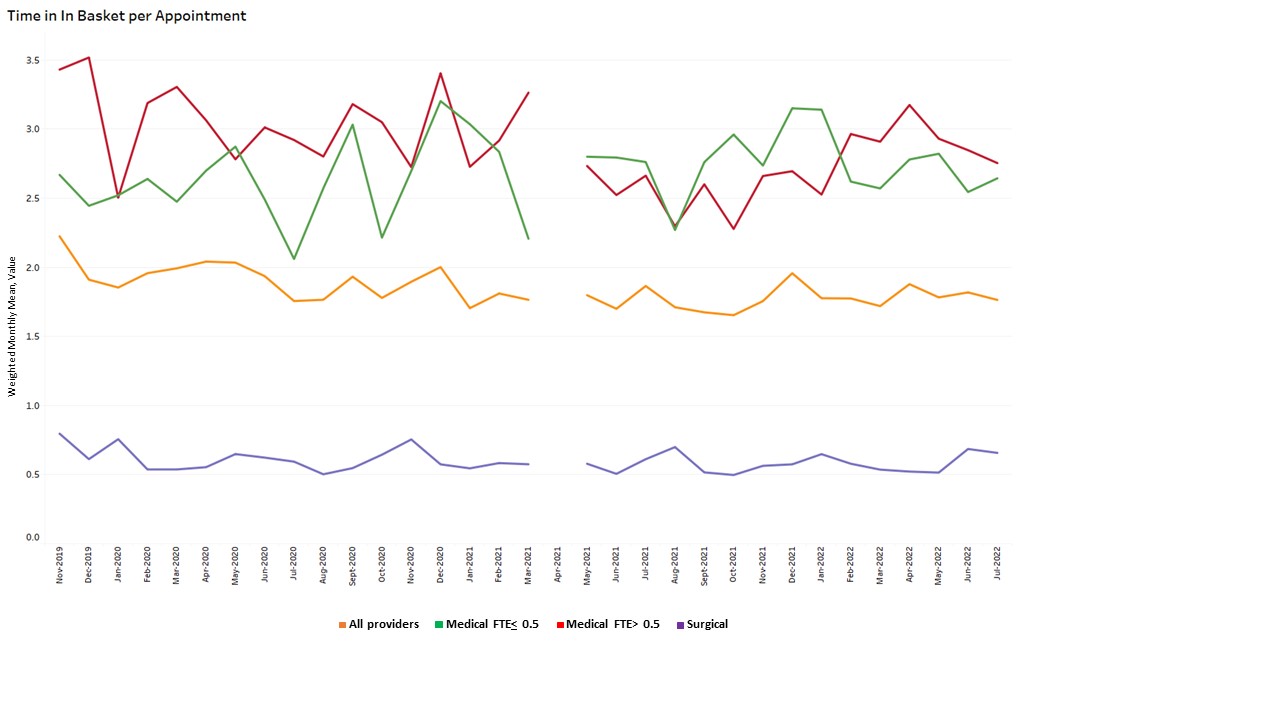


**Figure 7**. Time in In Basket per Appointment (group mean values).


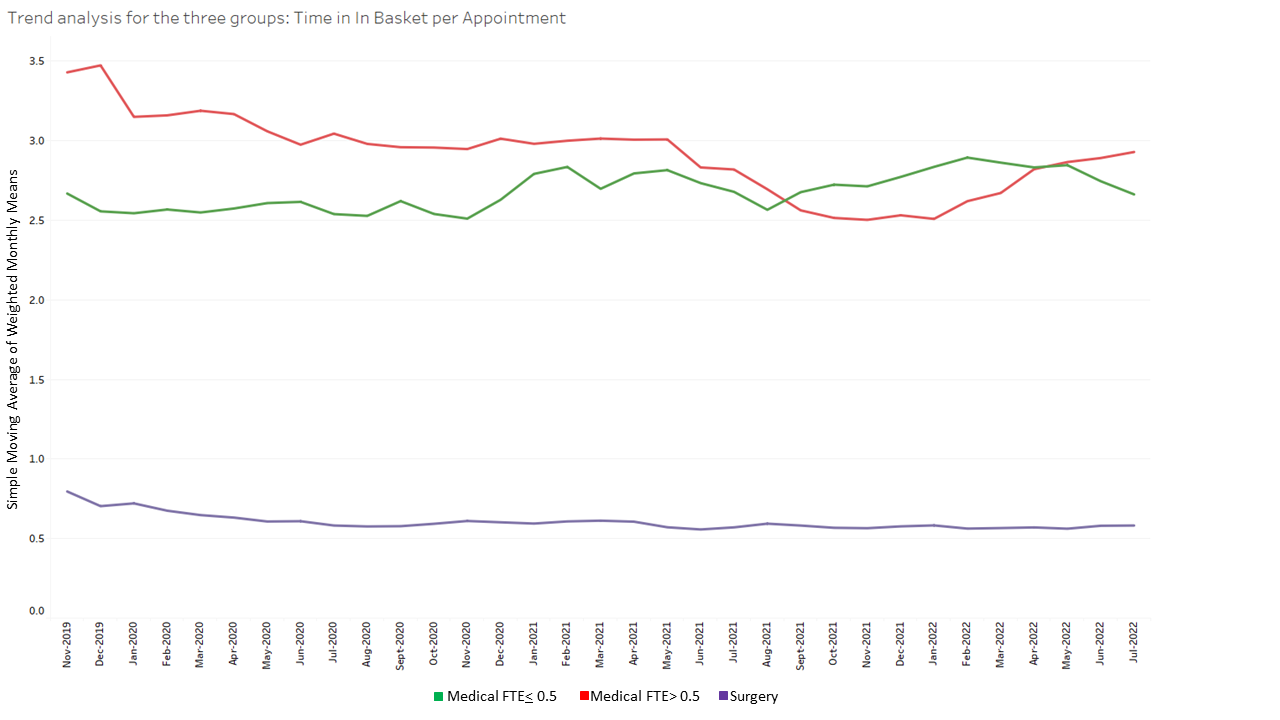


**Figure 8**. Time in In Basket per Appointment (SMA analysis by group).

## Turnaround time


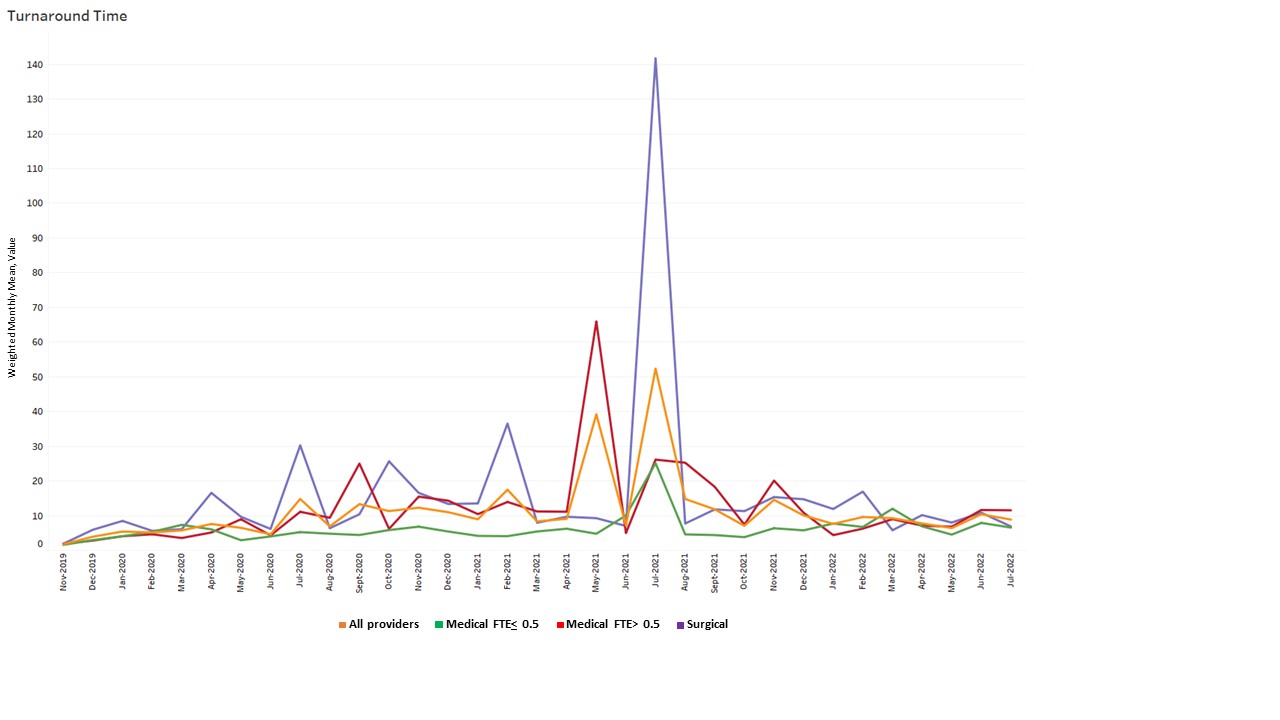


**Figure 9**. Turnaround Time (group mean values).


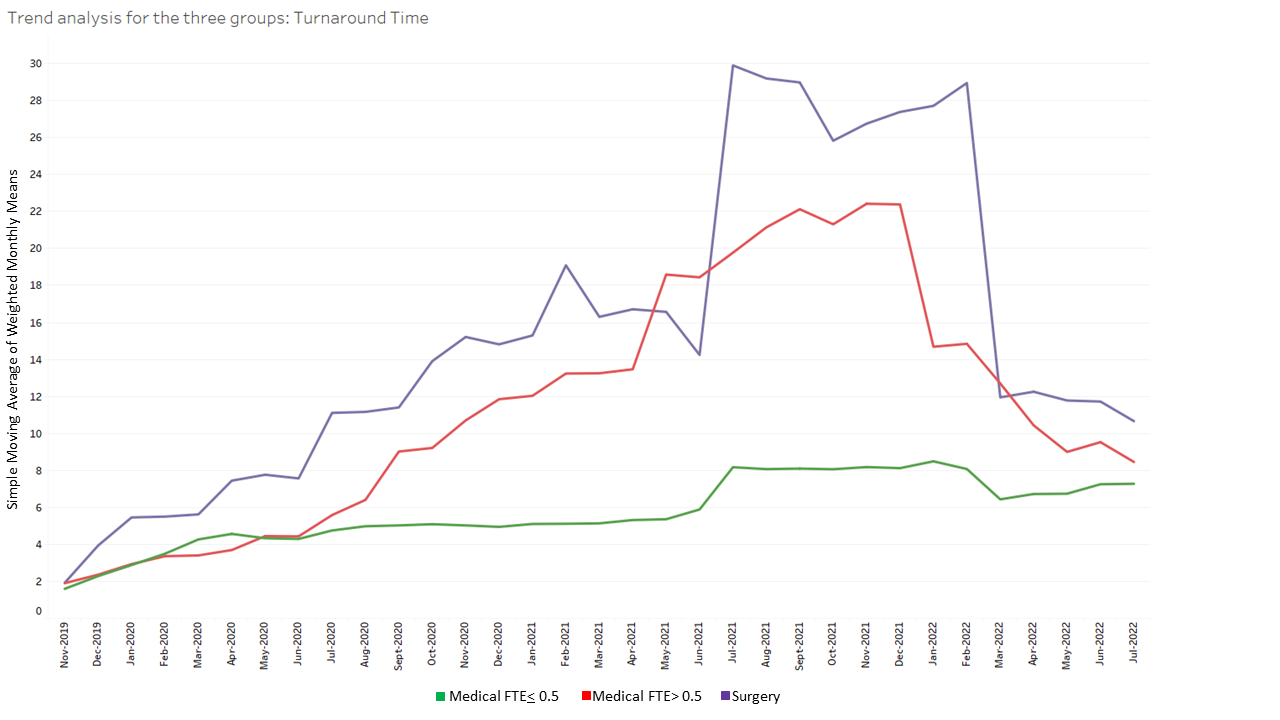


**Figure 10***.* Turnaround Time (SMA analysis by group).
